# Birt-Hogg-Dubé syndrome: A case series highlighting pulmonary manifestations, rare renal involvement and role of familial diagnosis

**DOI:** 10.5578/tt.202501987

**Published:** 2025-03-24

**Authors:** Berna AKINCI ÖZYÜREK, Kerem ENSARİOĞLU, Esma Sevil AKYURT, Tuğçe ŞAHİN ÖZDEMİREL, Göktürk FINDIK, Seçkin ÖZGÜL, Kayhan Çetin ATASOY, Mehmet Ali ERGÜN

**Affiliations:** 1 Clinic of Pulmonary Medicine, Ankara Atatürk Sanatorium Training and Research Hospital, Ankara, Türkiye; 2 Clinic of Thoracic Surgery, Ankara Atatürk Sanatorium Training and Research Hospital, Ankara, Türkiye; 3 DClinic of Gastroenterology, Ankara Atatürk Sanatorium Training and Research Hospital, Ankara, Türkiye; 4 Department of Radiology, Koç University Faculty of Medicine, Ankara, Türkiye; 5 Department of Medical Genetics, Gazi University Faculty of Medicine, Ankara, Türkiye

## Abstract

**ABSTRACT**

**
Birt-Hogg-Dubé syndrome: A case series highlighting pulmonary manifestations, rare renal involvement and role of familial diagnosis
**

*
Birt-Hogg-Dubé syndrome (BHDS) is a rare autosomal dominant disease characterized by cutaneous lesions and renal tumors, along with the presence of pulmonary cysts. In most cases, it is caused by a mutation in the folliculin (FLCN) gene. While normally evaluated as a triad, the isolated pulmonary presentation can still be expected, and repeated pneumothorax history should be evaluated for BHDS, especially if it is present in family members. In this case series, three patients with different clinical findings were reported. The first patient had a repeated pneumothorax history, along with angiomyolipoma, a relatively unexpected presentation of renal BHDS involvement. The incidental presence of pneumothorax in his mother had strengthened the diagnosis of BHDS, for which she also had renal cysts and additional cutaneous lesions. While these two patients were evaluated on an inpatient basis, the third patient, the son of the first patient, had been evaluated for BHDS presence, and pneumothorax, along with pulmonary cysts, was observed. All three patients were then later referred to a genetic center for confirmation of the FLCN mutation and tested positive for it. These case series illustrate the possibility of a different clinical presentation within the same family and at different ages, along with rare renal presentation and the possible asymptomatic indolent nature of the disease.
*

**Key words:** *Angiomyolipoma; autosomal dominant; Birt-Hogg-Dube syndrome; folliculin
*

**ÖZ**

**
Birt-Hogg-Dubé sendromu: Pulmoner semptomları, nadir böbrek tutulumunu ve ailesel geçisin rolünü vurgulayan bir vaka serisi
**

*
Birt-Hogg-Dubé sendromu (BHDS), pulmoner kistlerin varlığı ile birlikte kutanöz lezyonlar ve böbrek tümörleri ile karakterize nadir otozomal dominant bir hastalıktır. Çoğu vakada, bu duruma folikülin (FLCN) genindeki bir mutasyon neden olur. Normalde bir triad olarak değerlendirilse de izole akciğer tutulumu görülebilir ve tekrarlayan pnömotoraks öyküsü, özellikle aile bireylerinde de benzeri klinik mevcutsa hasta BHDS açısından değerlendirilmelidir. Bu olgu serisinde farklı klinik bulgulara sahip üç hasta sunulmaktadır. İlk hastada, böbrek BHDS tutulumunun nispeten beklenmedik bir sunumu olan anjiyomiyolipomun yanı sıra tekrarlayan bir pnömotoraks öyküsü vardı. Annesinde tesadüfen pnömotoraks görülmesi, annede böbrek kistleri ve ek deri lezyonlarının da bulunması ile BHDS tanısını güçlendirmişti. Bu iki hastaya ek olarak, birinci hastanın oğlu olan üçüncü hastada BHDS için yapılan değerlendirilmede akciğer kistlerinin yanı sıra pnömotoraks da gözlendi. Daha sonra üç hasta da FLCN mutasyonunun doğrulanması için genetik merkeze yönlendirildi ve test sonuçları pozitif çıktı. Bu vaka serileri, aynı aile içinde ve farklı yaşlarda farklı klinik belirtilerin ortaya çıkma olasılığının yanı sıra, nadir görülen böbrek belirtilerini ve hastalığın olası asemptomatik yavaş seyrini göstermektedir.
*

**Anahtar kelimeler:** *Anjiyomiyolipom; otosomal dominant; Birt-Hogg-Dube sendromu; folikülin
*

## INTRODUCTION

Birt-Hogg-Dubé syndrome (BHDS) is a rare autosomal dominant disease characterized by cutaneous lesions and renal tumors, along with the presence of pulmonary cysts (1). In most cases, it is caused by a mutation in the folliculin (FLCN) gene, first described in 1977 (2). The correlation between lung cysts and the syndrome was later clarified by Chung et al., and lung cysts’ characterization was better defined (3). The majority of the patients (90%) with BHDS have cysts present in computed tomography of the lung, and the incidence of pneumothorax is 50 times higher compared to the general population (4). In thirty percent of patients, the sole clinical presentation may be incidentally observed lung cysts (5). While some patients may be asymptomatic or with indolent symptoms, in elderly patients or with those having severe pulmonary involvement, dyspnea, coughing, and reoccurring pneumothorax may cause high morbidity (6). Pneumothorax incidence is especially high between the ages of 20 to 40 years, with some patients having years between pneumothorax attacks. In this case series, a family of three will be presented, among which BHDS diagnosis was eventually reached after clinical evaluation and genetic confirmation.

## Case Presentation

A 54-year-old male patient had been evaluated at the emergency ward with acute chest pain. The patient had a pneumothorax history five years ago and had no other known comorbidities. The former pneumothorax was treated by chest tube drainage and was accepted as spontaneous pneumothorax. The patient had severe left chest pain, with reduced

lung sounds on the same side, and had evident desaturation at room air with a finger oxygen saturation probe. Immediate chest tube drainage was performed on the patient, with the primary diagnosis being pneumothorax. After tube insertion and underwater drainage, air expulsion and clinical improvement were observed.

At the performed chest X-ray, radio-opacity was bilaterally present in both lung zones and was especially prominent in the lower left lung, with subpleural involvement (Figure 1). Upper zones were relatively spared, albeit relatively hyperlucent compared to other zones. This involvement of the left lung was attributed to the recent pneumothorax and

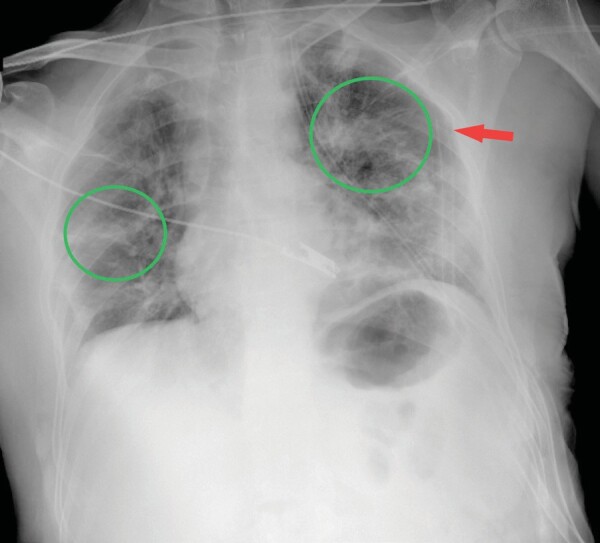

**Figure 1.** Fifty four-year-old male patient emergency admission chest X-ray.

In the given chest X-ray imaging, we may observe the placed chest tube (red arrow) as a treatment of pneumothorax and increased zones of density, which are circled in green.

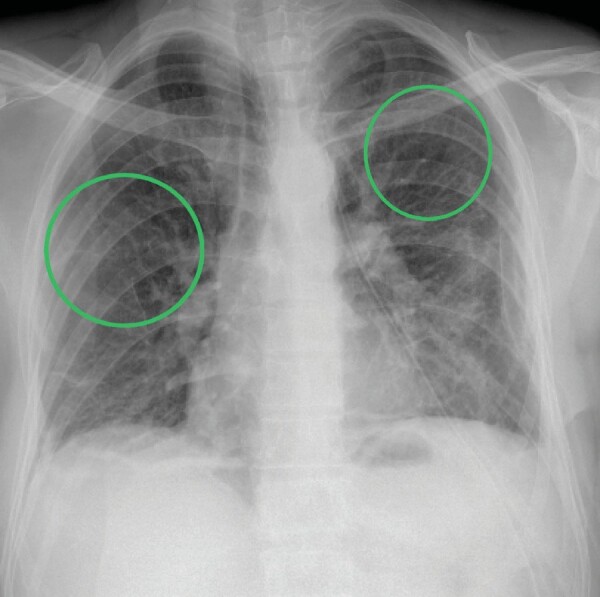

**Figure 2.** Treatment response after tube drainage.

Treatment response after tube drainage can be observed. Areas marked in green have significantly less density than the X-ray presented in figure 1.

chest tube drainage. The patient had an evident radiological response after the chest tube, and within a week, the chest drainage was ended with no latent morbidity present (Figure 2).

When questioned for detailed medical history, the patient stated that he had worked in a mechanic’s workshop for 21 years and stopped working there five years ago. His working conditions exposed him to sand dust, asbestos particles, and fine organic material dust. He did not have any smoking history, and at his former hospitalization for pneumothorax five years ago, he was evaluated for a possible hypersensitivity pneumonitis; the diagnosis was later excluded after bronchoscopy, radiological imaging, preserved respiratory function test results, and unsuitable clinic presentation. However, a point of interest was that the patient stated his mother had similar incidences of pneumothorax, with the two requiring chest tube drainage within three years. Upon further investigation, the patient had ultrasonography findings consistent with bilateral renal cysts; however, she did not apply for any follow-up.

Incidentally, this patient had to be admitted to the same thoracic surgery ward, as she, too, had symptoms consistent with pneumothorax two days

after the discharge of his son. Her initial chest X-ray favored a hyperlucent in the left upper lung, for which computed tomography was performed for detailed investigation (Figure 3). In the requested imaging, the patient had left-sided pneumothorax, which was mostly limited to the left upper lobe and lung cysts in the contralateral lobe (Figure 4).

The patient, while considered clinically stable and without any evident desaturation, still had to go through chest tube insertion due to the size of the pneumothorax. During routine physical examination, cutaneous lesions were observed, for which dermatology consultation was requested, and the lesions were defined as cutaneous hamartomas. The triad of renal cysts, reoccurring pneumothorax, and cutaneous lesions, along with familial history, had led to the diagnosis of BHDS. The requested renal ultrasonography imaging for the 54-year-old male patient confirmed renal involvement; however, the requested renal magnetic resonance imaging favored angiomyolipoma. The 74-year-old female patient was later discharged after subcutaneous emphysema resolved, which was attributed to the chest tube drainage procedure.

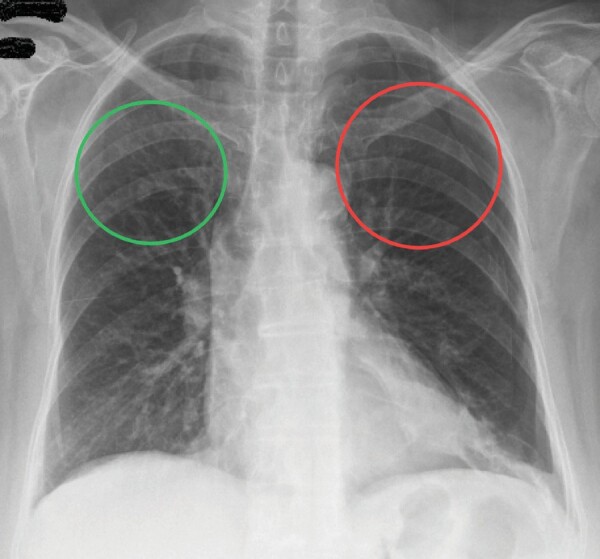

**Figure 3.** Seventy four-year-old female patient initial evaluation chest X-ray.

In the requested chest X-ray for initial evaluation, a clear differ- ence, in contrast, can be observed, with the left upper lung zone (circled in red) being hyper lucent, compared to the left upper lung zone (circled in green). This was considered in favor of either emphysema or air trapment, which in this case, was assumed to be a suspicious finding for possible air cysts or local- ized pneumothorax.

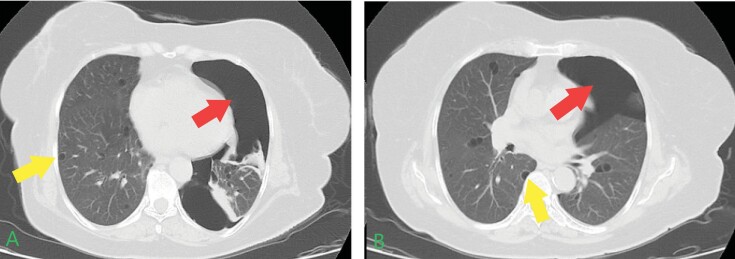

**Figure 4.** Seventy four-year-old female patient chest computed tomography findings.

In both imaging sections, **A-B**, pneumothorax is evident with more prominent findings in section **A**. Bilateral pulmonary cysts with varying diameters are also observed (red allows indicate pneumothorax, and yellow arrows indicate pulmonary cysts).

The 54-year-old initial patient had a son, for which he, too, was evaluated for the syndrome. While entirely asymptomatic with no specific dermatological and renal findings, along with a chest X-ray considered normal, this 29-year-old male patient was evaluated by a chest tomography for possible lung cysts (Figure 5). Chest tomography was consistent with the right upper lobe minimal pneumothorax and bilateral lung

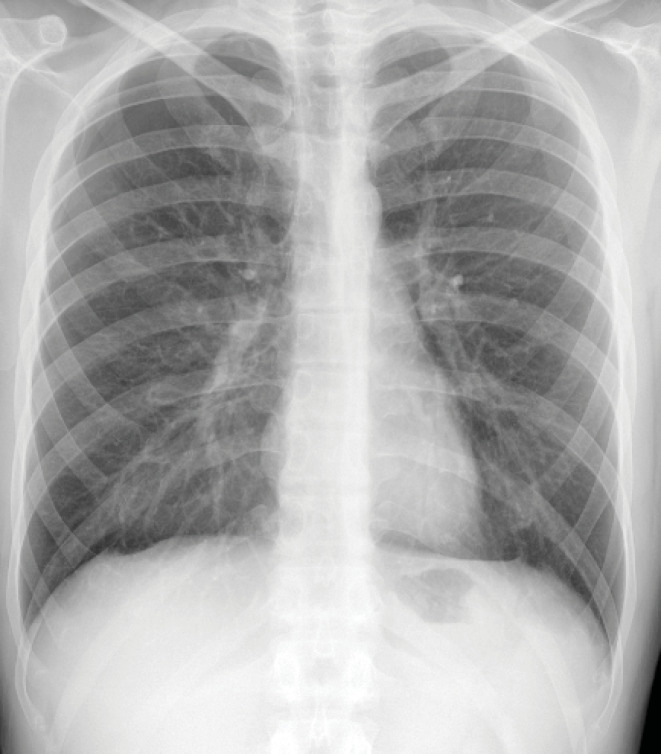

**Figure 5.** Twenty nine-year-old male patient initial evaluation chest X-ray.

In the requested chest X-ray for the 29-year-old male patient, no clear-cut abnormality could be observed.

cysts, which was considered a part of BHDS involvement (Figure 6). All three patients were then later referred to another hospital’s genetic center for the confirmation of diagnosis, and FLCN mutation was observed in all three patients. The patients were later put on a routine follow-up program. For a duration of four years, the patients are considered stable.

The 54-year-old patient was evaluated four years later in the emergency ward, this time with right pleuritic pain. As his and his familial history was well known, a chest X-ray was performed to exclude pneumothorax. In the requested imaging, evident pneumothorax was present in the right lung, with adequate response observed radiologically and clinically after tube drainage (Figure 7). Chest tomography was performed for further evaluation, and bilateral lung cysts were found (Figure 8). After treatment by drainage, the patient was considered stable for discharge in the first week of evaluation after admission to the thoracic surgery ward.

## DISCUSSION

BHDS is a rare syndrome with autosomal inheritance. This contributes to the possibility of early diagnosis if any other family members has been diagnosed earlier. As seen in our case series, the first patient’s symptoms, while nonspecific, were clarified after the evaluation of his relative, which led to the early diagnosis of the third asymptomatic family member. The original definition of BHDS consists of dermatological findings, as observed in these cases;

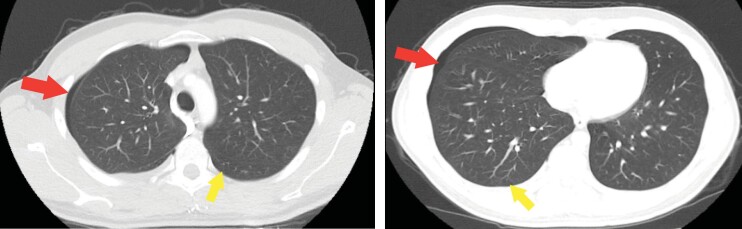

**Figure 6.** Twenty nine-year-old male patient chest tomography findings.

In both imaging sections, **A-B**, pneumothorax is evident, albeit at a reduced rate compared to the 54-years-old male patient. Bilateral pulmonary cysts with minimal diameters are also observed, with the size difference being noted compared to the 54-year-old male patient's imaging (red allows indicate pneumothorax, yellow arrows indicate pulmonary cysts).

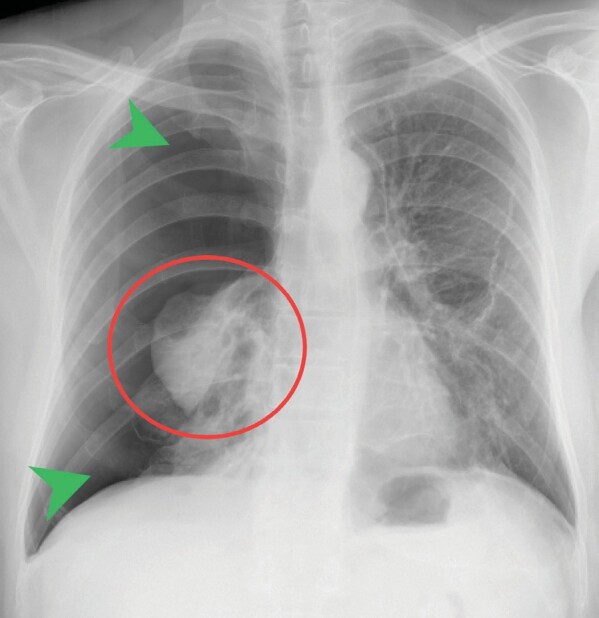
in some patients, with pulmonary cysts leading to possible pneumothorax. These cysts are often observed under 1 cm in size. Renal cancer, caused by a wide range of underlying tumor types, remains the most mortal manifestation of BHDS (10). These renal malignancy types include but are not limited to oncocytic tumors, clear cell carcinoma, and papillary carcinoma (11).

The presentation of angiomyolipoma in the initial patient, however, is a quite rare manifestation and is not generally expected in the list of commonly observed renal tumors of BHDS. This presentation confirms the diverse nature of BHDS, which was further explained in the study of Byrne et al., which attributes this finding to a similar protein pathway between BHDS and tuberous sclerosis complex mutations (12).

**Figure 7.** Fifty four-year-old male patient with reoccuring right side pneumothorax.

In the requested chest X-ray, the pneumothorax is clearly observ- able, with the lung itself reduced to the hilar area (red circle), and nearly all zones have lost their corresponding parenchymal imaging with only findings suitable of air density being observed (green arrows).

it is possible for the syndrome to be partially presented, such as with renal and pulmonary findings, or solely with one organ involvement (7,8).

Cutaneous lesions are the most common manifestation of BHDS, with fibrofolliculomas being the most frequently seen findings (9). Other skin lesions include trichodiscomas and acrochordons. Pulmonary manifestations, as stated, can be the sole manifestation

The diagnosis of BHDS is confirmed by multiple cutaneous findings, pulmonary cysts and spontaneous pneumothorax history, renal tumors, or any combination of these with familial and identification of a germline variant in FLCN by DNA sequencing. As reported in our cases, most patients and families often fulfill some of these criteria. Genetic testing confirms the diagnosis in the majority of cases, yet some patients may be diagnosed on clinicopathologic evaluation (13).

The relationship of BHDS to polyps and colorectal cancer was described early, but this association is not currently recognized (14). Nevertheless, FLCN gene mutations have recently been implicated in the development of colon cancer, questioning this

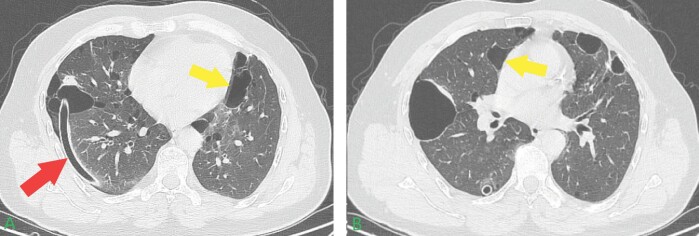

**Figure 8.** Fifty four-year-old male patient readmission chest tomography findings.

In the first tomography section, the longitudinal section of the chest tube can be more clearly observed (section **A**, red arrow). In both sections, **A** and **B**, yellow arrows indicate the presence of pulmonary cysts, increased in diameter and count compared to the initial findings.

viewpoint (15). Despite the fact that malignant colorectal pathology screening after BHDS diagnosis is recommended only if there is a family history of colorectal cancer, which was the case with our patient’s uncle, who had a history of rectum cancer. So, the patient underwent a screening colonoscopy, but no pathological findings were detected.

Currently, there are no definitive treatment modalities for BHDS. The primary approach consists of genetic counseling for the family due to the disease’s autosomal dominant nature and symptom control (8). In the case of fibrofolliculomas, local resection of cutaneous lesions may be performed. However, it is known to reoccur (9). Pulmonary manifestations cannot be often pre-emptively treated, with the treatment of whatever pathology at hand, such as chest tube for pneumothorax or pleurodesis in repeated cases, is the standard approach. Radiological evaluation is used for both follow-ups of pulmonary involvement and screening for possible renal tumors.

## CONCLUSION

In the case series, a patient and his family had been diagnosed with BHDS after the initial evaluation of the first patient. Every patient reported here had a different characteristic, with the 54-year-old patient having repeated pneumothorax history along with atypical renal involvement. The second, 74-year-old female patient had a more typical clinical presentation, with renal, dermatologic, and

pulmonary involvement consistent with the routinely expected BHDS triad. The final, 29-year-old patient was asymptomatic and yet had minimal pneumothorax in detailed chest imaging. When evaluated together, the case series presents a good estimation of the nature of BHDS, with varying clinical and radiological presentations. Genetic confirmation is utilized for confirmation of the disease, as reported in our study.

BHDS remains a rare case of pulmonary cysts and pneumothorax and should be kept in mind in patients with characteristic findings of BHDS and familial history of pneumothorax. Evaluation of other relatives, even those without any symptoms, should be performed, as pre-emptive diagnosis may benefit the diagnostic process in case of an emergency. Rare manifestations of renal pathologies are possible and, in the presence of two other main criteria, should warrant an investigation for BHDS.

## CONFLICT of INTEREST

The authors have no conflict of interest to declare.

## AUTHORSHIP CONTRIBUTIONS

Concept/Design: BAÖ, TŞÖ, GF, SÖ Analysis/Interpretation: KE, BAÖ, GF, SÖ, KÇA, MAE Data acqusition: BAÖ, ESA, SÖ, KÇA, MAE

Writing: KE, BAÖ, ESA

Clinical Revision: KE, BAÖ, TŞÖ Final Approval: All of authors

## REFERENCES

1. *Birt AR, Hogg GR, Dubé WJ. Hereditary multiple fibrofol- liculomas with trichodiscomas and acrochordons. Arch Dermatol 1977; 113: 1674-7.*
2. *
  Menko FH, van Steensel MAM, Giraud S, Friis-Hansen L, Richard S, Ungari S, et al. Birt-Hogg-Dubé syndrome: Diagnosis and management. Lancet Oncol 2009; 10: 1199-206. https://doi.org/10.1016/S1470-2045(09)70188-3*
3. *
  Chung JY, Ramos-Caro FA, Beers B, Ford MJ, Flowers F. Multiple lipomas, angiolipomas, and parathyroid adeno- mas in a patient with Birt-Hogg-Dube syndrome. Int J Dermatol 1996; 35: 365-7. https://doi.org/10.1111/j.1365-4362.1996.tb03642.x*
4. *
  Kumasaka T, Hayashi T, Mitani K, Kataoka H, Kikkawa M, Tobino K, et al. Characterization of pulmonary cysts in Birt- Hogg-Dubé syndrome: Histopathological and morpho- metric analysis of 229 pulmonary cysts from 50 unrelated patients. Histopathology 2014; 65: 100-10. https://doi.org/10.1111/his.12368*
5. *
  Kunogi M, Kurihara M, Ikegami TS, Kobayashi T, Shindo N, Kumasaka T, et al. Clinical and genetic spectrum of Birt- Hogg-Dube syndrome patients in whom pneumothorax and/or multiple lung cysts are the presenting feature. J Med Genet 2010; 47: 281-7. https://doi.org/10.1136/jmg.2009.070565*
6. *
  Tomassetti S, Carloni A, Chilosi M, Maffè A, Ungari S, Sverzellati N, et al. Pulmonary features of Birt-Hogg-Dubé syndrome: Cystic lesions and pulmonary histiocytoma. Respir Med 2011; 105: 768-74. https://doi.org/10.1016/j.rmed.2011.01.002*
7. *
  Schmidt LS, Nickerson ML, Warren MB, Glenn GM, Toro JR, Merino MJ, et al. Germline BHD-mutation spectrum and phenotype analysis of a large cohort of families with Birt-Hogg-Dubé syndrome. Am J Hum Genet 2005; 76: 1023-33. https://doi.org/10.1086/430842*
8. *
  Toro JR, Wei MH, Glenn GM, Weinreich M, Toure O, Vocke C, et al. BHD mutations, clinical and molecular genetic investigations of Birt-Hogg-Dubé syndrome: A new series of 50 families and a review of published reports. J Med Genet 2008; 45: 321-31. https://doi.org/10.1136/jmg.2007.054304*
9. *
  Aivaz O, Berkman S, Middelton L, Linehan WM, DiGiovanna JJ, Cowen EW. Comedonal and Cystic Fibrofolliculomas in Birt-Hogg-Dube Syndrome. JAMA Dermatol 2015; 151: 770-4. https://doi.org/10.1001/jamadermatol.2015.0215*
10. *
  Pavlovich CP, Walther MM, Eyler RA, Hewitt SM, Zbar B, Linehan WM, et al. Renal tumors in the Birt-Hogg-Dubé syndrome. Am J Surg Pathol 2002; 26: 1542-52. https://doi.org/10.1097/00000478-200212000-00002*
11. *
  Pavlovich CP, Grubb RL, Hurley K, Glenn GM, Toro J, Schmidt LS, et al. Evaluation and management of renal tumors in the Birt-Hogg-Dubé syndrome. J Urol 2005; 173: 1482-6. https://doi.org/10.1097/01.ju.0000154629.45832.30*
12. *
  Byrne M, Mallipeddi R, Pichert G, Whittaker S. Birt-Hogg- Dubé syndrome with a renal angiomyolipoma: Further evidence of a relationship between Birt-Hogg-Dubé syn- drome and tuberous sclerosis complex. Australas J Dermatol 2012; 53: 151-4. https://doi.org/10.1111/j.1440-0960.2011.00738.x*
13. *
  Schmidt LS, Linehan WM. Molecular genetics and clinical features of Birt-Hogg-Dubé syndrome. Nat Rev Urol 2015; 12: 558-69. https://doi.org/10.1038/nrurol.2015.206*
14. *
  Martínez-Pérez A, Santos-Alarcón S, Armañanzas-Villena E, Soriano-Camacho P. Birt-Hogg-Dubé syndrome and colon polyps. Revista de Gastroenterología de México (English Edition) 2016; 81: 53-4. https://doi.org/10.1016/j.rgmxen.2016.02.003*
15. *
  Sattler EC, Syunyaeva Z, Reithmair M, Dempke W, Steinlein OK. Colorectal cancer risk in families with Birt-Hogg-Dubé syndrome increased. Eur J Cancer 2021; 151: 168-74. https://doi.org/10.1016/j.ejca.2021.04.013*

